# Long-term remission after allogeneic hematopoietic stem cell transplantation in LPS-responsive beige-like anchor (LRBA) deficiency

**DOI:** 10.1016/j.jaci.2014.10.048

**Published:** 2015-05

**Authors:** Markus G. Seidel, Tatjana Hirschmugl, Laura Gamez-Diaz, Wolfgang Schwinger, Nina Serwas, Andrea Deutschmann, Gregor Gorkiewicz, Werner Zenz, Christian Windpassinger, Bodo Grimbacher, Christian Urban, Kaan Boztug

**Affiliations:** a Department of Pediatrics and Adolescent Medicine, Division of Pediatric Hematology-Oncology, Medical University Graz, Graz, Austria; d Department of Pediatrics and Adolescent Medicine, Division of General Pediatrics, Medical University Graz, Graz, Austria; e Institute of Pathology, Medical University Graz, Graz, Austria; f Institute of Human Genetics, Medical University Graz, Graz, Austria; b CeMM Research Center for Molecular Medicine of the Austrian Academy of Sciences, Vienna, Austria; c Center for Chronic Immunodeficiency, University Medical Center, Freiburg, Germany; g Department of Pediatrics and Adolescent Medicine, Medical University Vienna, Vienna, Austria

To the Editor:

LPS-responsive vesicle trafficking, beach and anchor containing protein (LRBA) deficiency has been identified as a primary immunodeficiency (PID) characterized by recurrent infections associated with autoimmunity, such as inflammatory bowel disease and autoimmune cytopenias (see Fig E1 in this article's Online Repository at www.jacionline.org).^1-3^ A wide range of immunosuppressive treatment measures have only induced temporary relief in affected subjects. Although allogeneic hematopoietic stem cell transplantation (HSCT) is the current treatment for many forms of PIDs, HSCT is less established in patients with autoimmune disease^4,5^ and has not yet been reported in LRBA-deficient patients.

We studied a consanguineous family of Kurdish origin with a systemic autoimmune disorder. Patient 1's symptoms started at 2 years of age with immune thrombocytopenia (ITP; Fig 1, *A*). Serum immunoglobulin concentrations were slightly increased, and the cellular immunophenotype was normal (Table I and see Table E1 in this article's Online Repository at www.jacionline.org). A lymph node biopsy performed because of generalized lymphoproliferative disease (LPD) revealed a follicular lymphatic hyperplasia with abundant (about 20% to 30%) CD3^+^ and CD4^−^ and CD8^−^ double-negative T lymphocytes (DNT cells; Fig 1, *C*), suggesting an immune dysregulation, lymphocyte maturation, or apoptosis defect compatible with autoimmune lymphoproliferative syndrome (ALPS).^6,7^ HSCT was performed with the clinically healthy HLA-identical mother as the donor (see the additional text in this article's Online Repository at www.jacionline.org), leading to complete remission with persisting full donor chimerism and without signs of acute or chronic graft-versus-host disease (GvHD). Four years after HSCT, ITP relapsed but responded well to high-dose intravenous immunoglobulin (IVIG) treatment. When romiplostim was started, platelet counts normalized, and administration of romiplostim (5 μg/kg, every 4 to 6 weeks) without further need for immunosuppression or IVIG has led to sustained but treatment-dependent remission.^8^

Patient 2, the now 11-year-old younger sister of patient 1, became symptomatic at 5 years of age (fulminant autoimmune hemolytic anemia; Fig 1, *B*). Immunosuppression was started immediately (corticosteroids, mycophenolate mofetil, and vincristine), leading to a sustained remission (Fig 1, *B*). Rituximab was administered (4 × 375 mg/m^2^; Fig 1, *B*) to secure the treatment response, especially given the severe course of her sister. Before treatment, immunoglobulin concentrations were mildly reduced (4.61 g/L IgG, normal IgA level, and 0.18 g/L IgM; Table I), direct and indirect Coombs test and platelet antibody results were positive, and DNT cell numbers were increased (3.4% of CD3^+^ cells), with an otherwise normal cellular immune phenotype (Table I and see Table E1), suggesting a familial ALPS-like disorder. Chronic enteropathy with increased calprotectin levels, borderline reduced elastase levels, and chronic norovirus positivity in stool were diagnosed. Gastroduodenoscopy specimens of patient 2 revealed inflammatory bowel disease, absence of plasma cells, and vasculitis (Fig 1, *C*; and see Fig E2, *D-I*, in this article's Online Repository at www.jacionline.org). She is being treated with budesonide and IVIG (1 g/kg body weight twice per month; trough level, 8-10 g/L) and requires parenteral nutrition (12-14 hours per night).

The fact that 2 patients born to consanguineous parents presented with a similar clinical phenotype prompted us to screen for an underlying (mono-) genetic defect. Homozygous intervals were mapped by applying the GeneChip Human-Mapping-250K-Nsp-Assay (Affymetrix, Santa Clara, Calif). Homozygous stretches were identified and overlaid with HomozygosityMapper.^9^ Both patients had identical homozygous intervals on chromosomes 2, 3, 4, 9, 11, and 15 (Fig 2, *A*). Exome sequencing and subsequent computational analysis of patient 1 revealed 23,582 exonic variants, of which 30 were rare missense, nonsense, or splice-site variants located inside the shared homozygous regions of the 2 siblings (see Table E2 in this article's Online Repository at www.jacionline.org). Among the final variant list, one frameshift deletion was identified, resulting in a premature stop codon. This mutation (NM_001199282:c.7162delA; p.T2388Pfs*7) is located inside the gene encoding LRBA. Sanger sequencing confirmed the presence and segregation of the variant, suggesting an autosomal recessive defect with full penetrance (Fig 2, *B*). Expression of the corresponding protein product was near absent (Fig 2, *C*).

Taken together, we describe a clinical, immunologic, and genetic analysis of 2 patients presenting with multiorgan autoimmunity and severe infections caused by a novel mutation in *LRBA*, the clinical spectrum of which both recapitulates and extends the previously described phenotypes (see additional text in this article's Online Repository).^1-3^ The fact that patient 1 had a profound immunodeficiency with life-threatening infections and refractory autoimmunity justified the approach of allogeneic matched family donor HSCT according to international guidelines.^4,5^ In our case allogeneic HSCT resulted in long-lasting partial remission in the patient with LRBA deficiency. The observation that mild autoimmune symptoms (ITP and vitiligo) have recurred in patient 1 years after HSCT despite full donor chimerism might be due to reduced LRBA expression compared with a healthy donor (in the same range as the heterozygous stem cell donor, who has detectable autoantibodies without clinical symptoms; see Fig 2, *C*, and additional text in this article's Online Repository), thus representing residual disease activity or late, limited chronic GvHD. These data show that HSCT might be a treatment option for patients with LRBA deficiency.

## Figures and Tables

**Fig E1:**
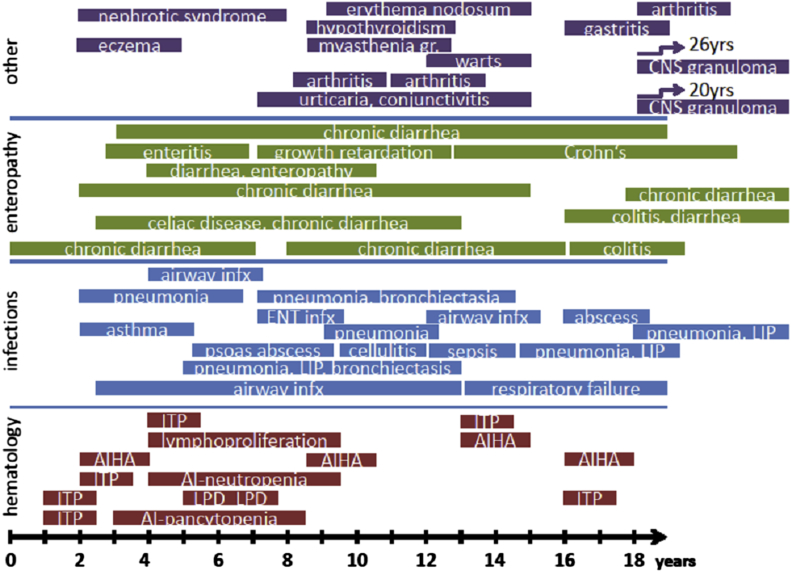
Spectrum and timely appearance of clinical symptoms of 11 previously published patients with LRBA deficiency. The time point (patient age) of the first documentation and, if possible, the end of a certain clinical condition of 11 children and young adults with LRBA deficiency are grouped according to the organ manifestation in “hematology,” “infections,” “enteropathy,” and “other” (from *bottom to top*), according to recent publications by Lopez-Herrera et al,^3^ Alangari et al,^1^ and Burns et al.^2^ Only the primary immune and autoimmune diseases, but not secondary symptoms, such as finger clubbing, or *cor pulmonale*, are shown. *AIHA*, Autoimmune hemolytic anemia; *AI-pancytopenia*, autoimmune pancytopenia; *CNS*, central nervous system; *ENT*, ear, nose, and throat; *infx*, infections; *ITP*, immune thrombocytopenia; *LIP*, lymphocytic interstitial pneumonitis; *LPD*, lymphoproliferative disease.

**Fig E2:**
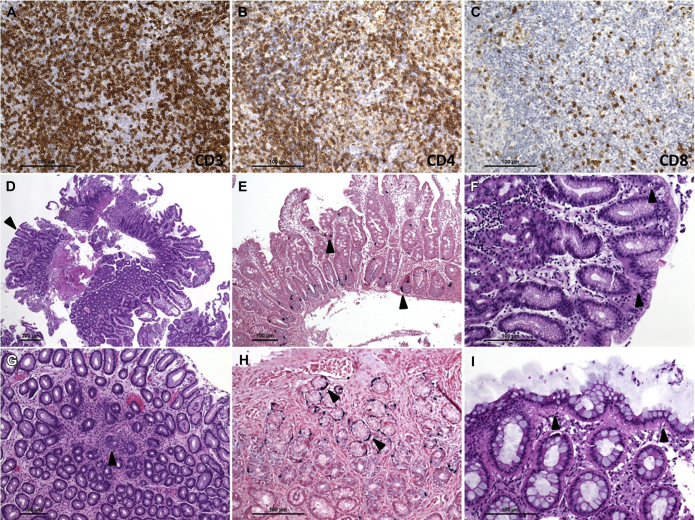
Histologic assessment of lymph node and gastrointestinal biopsy specimens. **A-C,** Consecutive lymph node sections showing an increased CD4/CD8 T-cell ratio. **D,** Duodenal biopsy specimens showing focal villous flattening. **E,** EBV RNA–positive enterocyte nuclei in duodenal biopsy specimens. **F,** Moderate chronic gastritis with apoptotic cell debris underneath the surface epithelium in the gastric corpus. **G,** Focally enhanced chronic active gastritis in the gastric antrum. **H,** EBV RNA–positive antral epithelial cells. **I,** Apoptotic cell debris beneath the surface epithelium in the colonic mucosa. Plasma cells were absent in all sections.

**Table E1:** Additional laboratory parameters of 2 patients with LRBA deficiency

	Patient 1		Patient 2	
	Before HSCT	After HSCT	Before rituximab	After rituximab
Humoral immune system	99.98% donor chimerism‖¶¶∗∗∗		(IVIG substituted)	
Anti–tetanus toxoid antibody	Good response‡	ND	NA	NA
Anti–*Haemophilus influenzae* B polysaccharide antibody	Good response‡	ND	NA	NA
IgG against EBV	NA	ND	Negative¶	NA
IgG against EBNA1	NA	ND	NA	NA
IgG against CMV	Negative∗	ND	208 U/mL¶	NA
IgG against hepatitis B	**Negative**∗	ND	NA	NA
Polio IgG	Good response‡	ND	NA	NA
ANCA	Negative/unspecific∗	Negative/unspecific‖	Negative‡‡‡	Negative‡‡‡
TPO antibody	ND	Negative¶¶	Negative‡‡‡	Negative‡‡‡
TR antibody	ND	Negative¶¶	Negative‡‡‡	Negative‡‡‡
TG antibody	**Positive**† (negative)	Negative¶¶	Negative‡‡‡	Negative‡‡‡
Islet cell antibody	**Positive**† (negative)	Negative¶¶	**Positive**†	Negative‡‡‡
Insulin antibody	ND	ND	Negative†	Negative‡‡
Glutamate decarboxylase antibody	ND	Negative§	ND	**11.9 U/L**‡‡ (0-9.5 U/L)
Tyrosine phosphatase antibody	ND	ND	ND	Negative‡‡
21-Hydroxylase antibody	ND	ND	ND	Normal‡‡
Gliadin IgA antibody	**150**† (<25)	ND	ND	Negative††
Gliadin IgG antibody	**25**† (<25)	ND	ND	Negative††
Transglutaminase antibody	ND	ND	ND	Negative††
Endomysial IgG antibody	Negative†	ND	ND.	Negative††
Laboratory chemistry (selected parameters)				
TSH	Normal‡‡‡	Normal‡‡‡	Normal‡‡‡	**6.56 μU/mL**†† (0.1-4 μU/mL)
fT4	Normal‡‡‡	Normal‡‡‡	Normal‡‡‡	15.1 pmol/L†† (9.5-24 pmol/L)
Elastase/stool (μg E1/g)	**22-88**∗† (200-2500)	ND	ND	**141-156**§§ (200-2500)
Calprotectin/stool (μg/g)	ND	ND	**272**∗ (0-100)	**1331**‡‡ (0-100)
Vitamin B12 (pg/mL)	ND	ND	**285**∗∗ (180-1100)	179†† (180-1100)
Vitamin A	ND	ND	ND	**23 μg/dL**§§ (30-70 μg/dL)
Vitamin D3	ND	ND	ND	**5.6 ng/mL** (30-60 ng/mL)††
Vitamin E	ND	ND	ND	**7 μmol/L**§§ (12-46 μmol/L)
Prothrombin time	Normal‡‡‡	Normal‡‡‡	Normal‡‡‡	**57%**‡‡ (70% to 120%)
Cellular immune system				
TREC copies/10^5^ CD3^+^CD45^+^ cells	ND	**0**¶¶ | **0**∗∗∗	ND	2.13‖‖
Vβ spectratyping (diversity in CD4^+^ and CD8^+^ T cells)	ND	CD4^+^ normal; mildly reduced in CD8^+^∗∗∗	ND	Normal in CD4^+^; mildly reduced in CD8^+^‖‖
Phagocyte function (oxidative burst, phagocytosis; *Escherichia coli*, DHR)†††	Normal∗	ND	ND	ND
Apoptosis assay (PHA- or IL-2–activated T cells; annexin V staining)†††	Normal∗ (anti-CD95)	ND	ND	Normal§§ (IL-2 withdrawal)
ADA activity	Normal∗	ND	ND	ND
PNP activity	Normal∗	ND	ND	ND
Genetic analyses done before performance of whole-exome sequencing				
*CD95*, *CD95L*, caspase 8, caspase 10	Normal†		Normal#	
*CTLA4* SNPs, *SAP*, *XIAP*	Normal†		Normal#	
*HLA-DQ8*	Positive		Positive	

Footnotes indicate time point of analysis. Pathologic results are shown in boldface (normal ranges are in parentheses). *ADA*, Adenosine desaminase; *ANCA*, anti-neutrophil cytoplasmic antibodies; *CMV*, cytomegalovirus; *DHR*, dihydrorhodamine; *fT4*, free thyroxine; *NA*, not applicable under IVIG substitution and not done before IVIG; *ND*, not done; *PNP*, purine nucleoside phosphorylase; *SAP*, signaling lymphocytic activation molecule (SLAM)-associated protein; *SNP*, single nucleotide polymorphism; *TG*, thyreoglobulin; *TPO*, thyroid peroxidase; *TR*, thyroid stimulating hormone receptor antibody; *TSH*, thyroid stimulating hormone; *XIAP*, X-linked inhibitor of apoptosis.

∗ At 6 years of age.

† At 8 years.

‡ Low/negative after a break of repetitive IVIG therapy at 6 years; good response/high normal values after one booster vaccination at 6.5 years of age.

§ At 10 years.

‖ At 14 years (4 years after stem cell transplantation).

¶ At 17 months.

# At 2 years.

∗∗ At 5.5 years before immunosuppression/rituximab.

†† At 7.5 years of age.

‡‡ At 9 years of age.

§§ At 10 years of age.

‖‖ At 11 years of age.

¶¶ At 19 years of age (9 years after HSCT).

∗∗∗ At 19.5 years of age (10 years after stem cell transplantation).

††† See the Methods section in this article's Online Repository.

‡‡‡ At repeated occasions.

**Table E2:** List of candidate mutations identified in the index patient

	Function	Gene	Protein name	Exonic function	Amino acid change	dbSNP137
1	Exonic	*LRBA*	LPS-responsive vesicle trafficking, beach and anchor containing	Frameshift deletion	NM_001199282:c.7162delA:p.T2388fs	
2	Exonic	*LRBA*	LPS-responsive vesicle trafficking, beach and anchor containing	Nonsynonymous SNV	NM_001199282:c.A2444G:p.N815S	rs140666848
3	Exonic	*PAPSS1*	3′-Phosphoadenosine 5′-phosphosulfate synthase 1	Nonsynonymous SNV	NM_005443:c.C997T:p.R333C	rs35176475
4	Exonic	*FAT4*	FAT atypical cadherin 4	Nonsynonymous SNV	NM_024582:c.C6970T:p.R2324W	
5	Exonic	*FAT4*	FAT atypical cadherin 4	Nonsynonymous SNV	NM_024582:c.G9577A:p.V3193I	rs143764643
6	Exonic	*MAML3*	Mastermind-like 3 (Drosophila)	Nonframeshift deletion	NM_018717:c.2302_2304del:p.768_768del	rs5862430
7	Exonic	*SLC10A7*	Solute carrier family 10, member 7	Nonsynonymous SNV	NM_001029998:c.T806C:p.V269A	
8	Exonic	*WNK2*	WNK lysine deficient protein kinase 2	Nonsynonymous SNV	NM_006648:c.A83T:p.E28V	
9	Exonic	*C9orf129*	Chromosome 9 open reading frame 129	Nonsynonymous SNV	NM_001098808:c.C359T:p.A120V	rs4744219
10	Exonic	*C9orf129*	Chromosome 9 open reading frame 129	Nonsynonymous SNV	NM_001098808:c.G274A:p.G92S	rs3122944
11	Exonic	*NUTMF*	NUT family member 2F	Nonframeshift deletion	NM_017561:c.2071_2073del:p.691_691del	rs150455117
12	Exonic	*NUTMF*	NUT family member 2F	Nonsynonymous SNV	NM_017561:c.C410G:p.S137C	rs202099818
13	Exonic	*FOXE1*	Forkhead box E1 (thyroid transcription factor 2)	Nonframeshift deletion	NM_004473:c.511_516del:p.171_172del	
14	Exonic	*COL15A1*	Collagen, type XV, alpha 1	Nonsynonymous SNV	NM_001855:c.A3002G:p.K1001R	rs35544077
15	Splicing	*ABCA1*	ATP-binding cassette, sub-family A (ABC1), member 1		NM_005502:exon41:c.5383-2->TTT	
16	Splicing	*SVEP1*	Sushi, von Willebrand factor type A, EGF and pentraxin domain containing 1		NM_153366:exon47:c.10505-2->T	
17	Exonic	*DNAJC25*	DnaJ (Hsp40) homolog, subfamily C, member 25	Nonsynonymous SNV	NM_001015882:c.T486G:p.F162L	
18	Splicing	*OLFML2A*	Olfactomedin-like 2A		NM_182487:exon3:c.462+1G>T	
19	Exonic	*RABEPK*	Rab9 effector protein with kelch motifs	Nonsynonymous SNV	NM_005833:c.C217T:p.H73Y	rs1128362
20	Exonic	*FAM73B*	Family with sequence similarity 73, member B	Nonsynonymous SNV	NM_032809:c.T299C:p.V100A	rs11544968
21	Exonic	*USP20*	Ubiquitin specific peptidase 20	Nonframeshift deletion	NM_001008563:c.1072_1074del:p.358_358del	rs10602985
22	Exonic	*PPAPDC3*	Phosphatidic acid phosphatase type 2 domain containing 3	Nonsynonymous SNV	NM_032728:c.G26A:p.R9H	rs148406586
23	Exonic	*GTF3C4*	General transcription factor IIIC, polypeptide 4, 90kDa	Nonsynonymous SNV	NM_012204:c.G10A:p.A4T	rs143172300
24	Exonic	*GOLGA6L10*	Golgin A6 family-like 10	Nonsynonymous SNV	NM_001164465:c.G1025A:p.R342Q	rs201670904
25	Exonic	*GOLGA6L10*	Golgin A6 family-like 10	Nonsynonymous SNV	NM_001164465:c.G1007A:p.R336Q	rs200685620
26	Exonic	*ALKBH3*	alkB, alkylation repair homolog 3	Nonsynonymous SNV	NM_139178:c.C684G:p.D228E	rs1130290
27	Splicing	*CREB3L1*	cAMP responsive element binding protein 3-like 1		NM_052854:exon12:c.1524-1->G	rs79068197
28	Exonic	*ACP2*	Acid phosphatase 2, lysosomal	Nonsynonymous SNV	NM_001610:c.T1177C:p.F393L	rs145420520
29	Exonic	*ALPK3*	Alpha-kinase 3	Nonsynonymous SNV	NM_020778:c.G4289A:p.R1430Q	rs150023454
30	Exonic	*ACAN*	Aggrecan	Nonsynonymous SNV	NM_001135:c.G1274A:p.G425E	

*SNV*, Single nucleotide variant.

**Fig 1:**
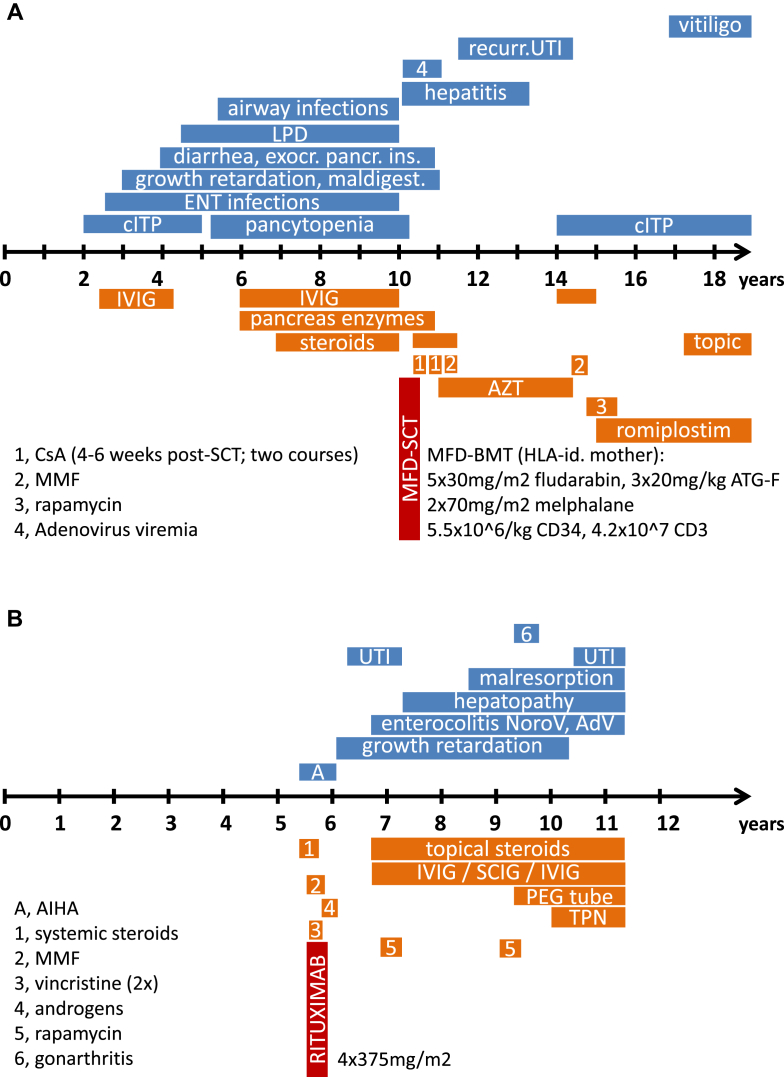

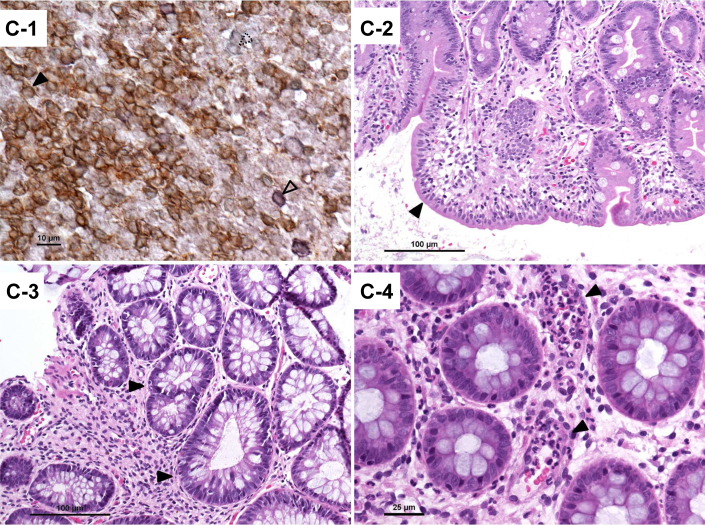
Clinical course of a familial autoimmunity syndrome caused by LRBA deficiency, immunohistochemical analysis of lymph node specimens (patient 1), and histologic assessment of gastrointestinal biopsy specimens (patient 2). **A,** Clinical course of a now 19-year-old girl, patient 1, including treatment and HSCT at the age of 10 years. **B,** Symptoms and treatment outline of patient 2. **C,***C.1*, Triple immunohistochemical staining of T-cell markers showing increased double-negative T-cell numbers marked only with an antibody against CD3 (*light blue/gray*, *dashed arrow*), which is reminiscent of CD95 deficiency; CD4^+^ (*brown*, *solid arrow*) and CD8^+^ (*purple*, *open arrow*) T cells are also shown. *C.2*, Duodenal biopsy specimens showing focal villous flattening and intraepithelial lymphocytosis. *C.3*, Colon mucosa with moderate crypt distortion and sparse apoptotic bodies. *C.4*, Signs of vasculitis indicated by abundant neutrophilic granulocytes within and migrating through the lamina propria capillaries of the colon mucosa. Plasma cells were absent in all sections. *AdV*, Adenovirus; *AIHA*, autoimmune hemolytic anemia; *ATG-F*, anti-thymocyte globulin-Fresenius (Fresenius Medical Care, Vienna, Austria); *AZT*, azidothymidine; *cITP*, chronic immune thrombocytopenia; *CsA*, cyclosporin A; *ENT*, ear, nose, and throat; *IVIG*, intravenous immunoglobulin subsitution; *LPD*, lymphoproliferative disease; *MFD-BMT*, matched family donor bone marrow transplantation; *MMF*, mycophenolate mofetil; *PEG tube*, percutaneous enterogastral tube; *TPN*, total parenteral nutrition; *SCIG*, subcutaneous immunoglobulin subsitution; *UTI*, urinary tract infection.

**Fig 2:**
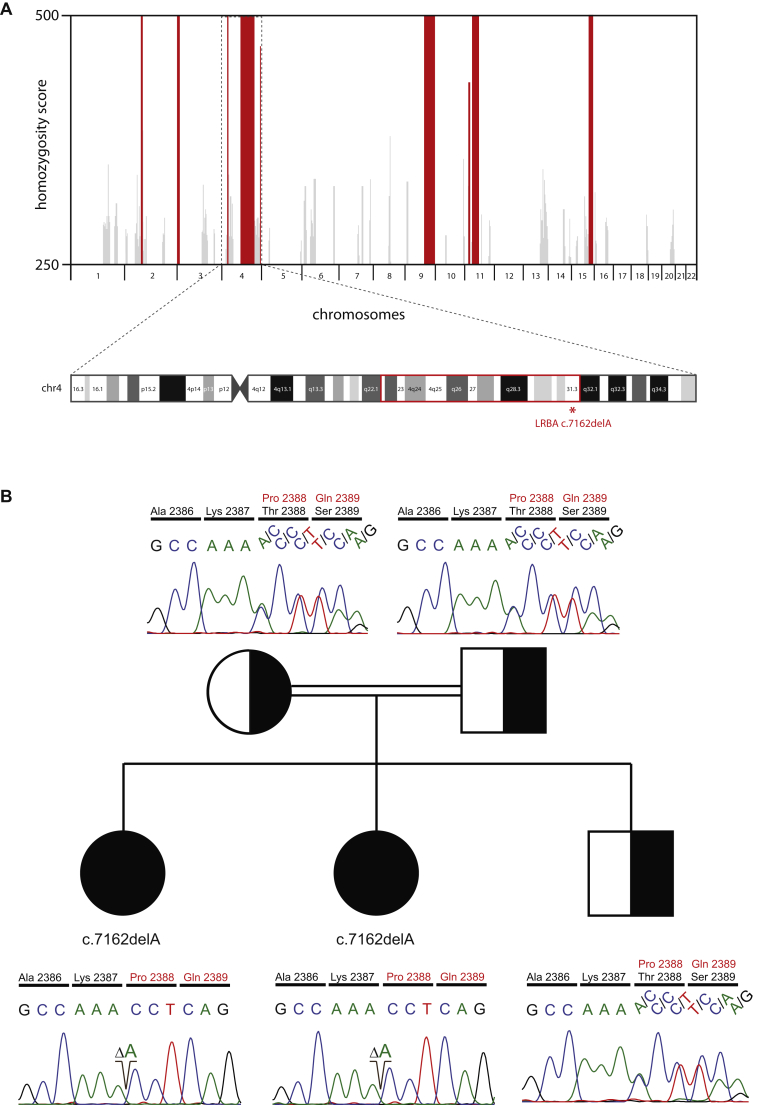

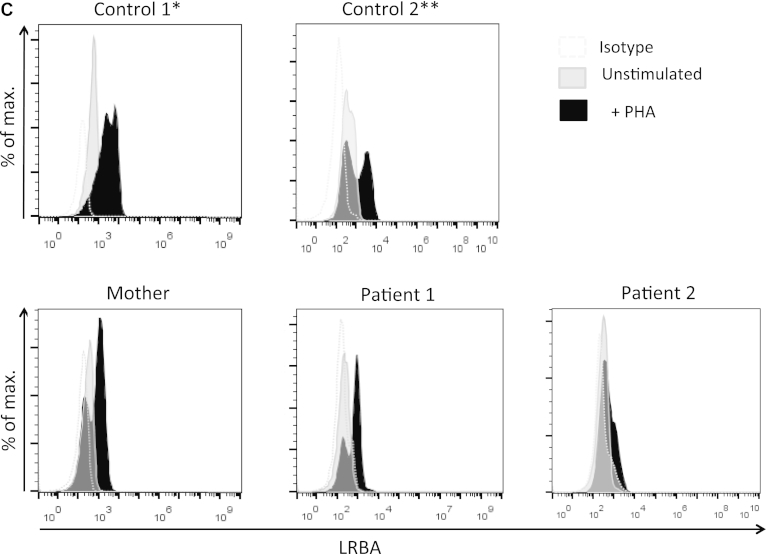
Representative depiction of single nucleotide polymorphism array–based homozygosity mapping and Sanger validation, pedigree of the core family, and LRBA protein detection by using fluorescence-activated cell sorting analysis. **A,** Chromosomal positions are plotted against the homozygosity score in a bar chart, with *red bars* indicating homozygous regions present in both affected siblings *(top)*. The disease-causing mutation is localized in a homozygous interval (q22.2-q31.3) on the long arm of chromosome 4, as emphasized by the *red box (bottom)*. **B,** Perfect segregation of the single base deletion (c.7162delA; p.T2388fs) is shown in the 2 patients, the nonaffected sibling, and the parents. *Solid symbols* indicate homozygous affected subjects, and *half-filled symbols* refer to the heterozygous carrier. Male and female subjects are distinguished by *squares* and *circles*, respectively. **C,** PBMCs were stimulated with PHA, as described in the Methods section in this article's Online Repository at www.jacionline.org. The increased LRBA protein expression after stimulation (*black*) compared with that in unstimulated cells (*gray*) is shown in the in-house control and in a travel control (1 and 2 asterisks, respectively; *upper panel*); is reduced in the LRBA-heterozygous mother, who was the stem cell donor, in patient 1 after HSCT; and is absent in patient 2 *(lower panel)*. The plot is representative of 2 independent analyses.

**Table I:** Laboratory parameters of 2 patients with LRBA deficiency

	Patient 1		Patient 2	
	Before HSCT	After HSCT	Before rituximab	After rituximab
Humoral immune system	99.98% donor chimerism‖¶¶∗∗∗		(IVIG substituted)	
IgG (g/L)	**16.7** (6.5-14.1)∗	11.3 (7-16)∗∗∗	**4.61** (5.5-12)∗∗	NA
IgG_1_ (g/L)	9.1 (3.5-9.1)∗	7.85 (4.05-10.11)∗∗∗	NA	NA
IgG_2_ (g/L)	3.06 (0.85-3.30)∗	3.69 (1.69-7.86)∗∗∗	NA	NA
IgG_3_ (g/L)	**1.83** (0.2-1.04)∗	**0.879** (0.11-0.85)∗∗∗	NA	NA
IgG_4_ (g/L)	0.01 (0.03-1.58)∗	0.481 (0.03-2.01)∗∗∗	NA	NA
IgA (g/L)	1.24 (0.83-2.17)∗	2.61 (0.7-4.0)∗∗∗	0.28 (0.21-2.92)∗∗	**<0.08** (0.31-3.06)‖‖
IgM (g/L)	1.43 (0.55-2.10)∗	1.64 (0.4-2.3)∗∗∗	**0.18** (0.37-1.41)∗∗	**0.02** (0.47-1.0)‖‖
IgE (kU/L)	24.7∗ (<110)	44 (0-100)∗∗∗	<19‡‡‡	<19‡‡‡
Autoimmunity (selected autoantibodies)				
Coombs test, direct	**1:64**∗	Negative∗∗∗	**Positive, 1:16-64**¶#∗∗	Negative††
Coombs test, indirect	**Positive**∗	Negative∗∗∗	**Positive**¶#	Negative††
Anti-platelet antibodies	**Positive**†	Positive‖ | negative∗∗∗	**Positive**#	Negative††
ANA	Negative‡‡‡	Negative‡‡‡	Negative‡‡‡	Negative‡‡‡
dsDNA antibody	Negative‡‡‡	Negative‡‡‡	Negative‡‡‡	Negative‡‡‡
Cardiolipin IgG antibody (U/mL)	**14**∗ (0-10)	**33**§ | 2.3∗∗∗ (0-10)	Negative‡‡‡	Negative‡‡‡
SMA (U/mL)	**50**∗ (negative)	**200**§ | negative∗∗∗ (negative)	Negative‡‡‡	Negative‡‡‡
AMA	Negative∗	**Positive,**§∗∗∗ (negative)	Negative‡‡‡	**Positive**†† | negative‡‡‡
M2 antibody## (U/mL)	Negative∗	**45.6**‖**| 88.0**∗∗∗ (0-5)	Negative‡‡‡	Negative‡‡‡
Cellular immune system				
CD3^+^ T cells/μL	1,930† (700-4,200)	736∗∗∗ (700-2,100)	2,799# (1,400-8,000)	1325§§ (700-4,200)
CD3^+^CD4^+^ cells/μL	1,511† (300-2,000)	324∗∗∗ (300-1,400)	2,010# (900-5,500)	931§§ (300-2,000)
CD3^+^CD8^+^ cells/μL	296† (300-1,800)	367∗∗∗ (200-900)	601# (400-2,300)	325§§ (300-1,800)
CD45RA^+^CD4^+^CD3^+^ cells (% of CD3^+^CD4^+^ cells)	64† (>15%)	**8.5**¶¶ | **6**∗∗∗ (>10%)	57# (>15)	26§§ (>10%)
αβTCR^+^CD3^+^ cells/μL	ND	638‖	2,578#	1,097§§
γδTCR^+^CD3^+^ cells/μL	ND	10‖	137#	39§§
αβTCRCD3^+^CD4^−^CD8^−^ (DNT cells [% of CD3^+^ cells])	0.95% to **3%**∗† (<2)	0.03%∗∗∗ (<2)	0.4%# (<2)	**3.24%**§§ (<2)
CD3^−^CD56^+^ NK cells/μL	108† (90-900)	**140**∗∗∗ (200-300)	291# (100-1,400)	81§§ (90-900)
iNKT cells Va24Vb11 (% of CD3^+^ cells)	ND	0.1%¶¶ (>0.01)	ND	0.02%‖‖ (>0.01)
CD19^+^ B cells/μL	335-**118**∗† (200-1,600)	213¶¶ (100-500)	413# (200-2,100)	**32**†† (200-1,600)
CD19^+^IgD^+^CD27^+^ cells (% of CD19^+^ cells)	ND∗†	**0.38**‖ | 30¶¶ (>2)	ND#	**0.02**†† (>2)
CD19^+^IgD^−^CD27^+^ cells (% of CD19^+^ cells)	ND∗†	**0.08**‖ | 31¶¶ (>2)	ND#	**0.00**†† (>2)
Lymphocyte stimulation *in vitro* detected based on tritiated thymidine incorporation (trigger and antigens in parentheses)†††	Normal† (PHA, SEB, CD3, PMA/ionomycin)	ND	ND	Normal‡‡ unstimulated: 1,716 cpm (1,650-7,162 cpm) PHA: 16,513 cpm (14,218-39,235 cpm) Concanavalin A: 11,384 cpm (4,928-29,519 cpm) CD3/CD28: 17,746 cpm (12,181-31,490 cpm)

Footnotes indicate time point of analysis. Pathologic results are shown in boldface (normal ranges are shown in parentheses). *AMA*, Anti-mitochondrial antibodies; *ANA*, antinuclear antibody; *dsDNA*, double-stranded DNA; *iNKT*, invariant natural killer T; *NA*, not applicable under IVIG substitution and not done before IVIG; *ND*, not done; *NK*, natural killer; *PHA*, phytohemagglutinine; *PMA*, phorbol 12-myristate 13-acetate; *SCT*, stem cell transplantation; *SEB*, staphylococcal enterotoxin B; *SMA*, smooth muscle autoantibodies; *TCR*, T-cell receptor.

∗ At 6 years of age.

† At 8 years.

§ At 10 years.

‖ At 14 years (4 years after stem cell transplantation).

¶ At 17 months.

# At 2 years.

∗∗ At 5.5 years before immunosuppression/rituximab.

†† At 7.5 years of age.

‡‡ At 9 years of age.

§§ At 10 years of age.

‖‖ At 11 years of age.

¶¶ At 19 years of age (9 years after HSCT).

## Subfraction of antimitochondrial antibodies directed against the M2 fraction of liver cell mitochondrial antigens located on inner mitochondrial membranes (comprising proteins of the 2-oxo-acid dehydrogenase complex).

∗∗∗ At 19.5 years of age (10 years after stem cell transplantation).

††† See the Methods section in this article's Online Repository.

‡‡‡ On repeated occasions.

## Appendix

Within the last 10 to 15 years, the paradigm of PIDs defined as inborn errors of immune cells causing a dangerous predisposition toward infections has been extended to include immune dysregulation syndromes and autoimmunity. This development was based on the identification of defects in regulatory cell subsets, such as forkhead box P3–positive and other regulatory T cells^E1,E2^; perturbed T- and B-cell maturation and tolerance checkpoint defects^E3,E4^; and dendritic cell–and phagocyte-mediated immune regulation.^E5,E6^ This is reflected by the modification of diagnostic criteria and the most recent classification of PIDs.^E7-E10^ LRBA protein deficiency was identified as a novel human disease gene causing PIDs with associated autoimmunity, including enteropathy and other autoimmune symptoms.^E11-E13^ However, the functional role of LRBA has remained largely elusive.

LRBA was initially identified as an LPS-inducible gene in B cells and macrophages^E14^ and is comprised of a “beige and Chediak-Higashi” (BEACH) domain, a pleckstrin homology and a WD40 repeat domain, which collectively are likely required for protein-protein and DNA-protein interactions.^E14,E15^ It has been shown that the BEACH-WD40 domains of LRBA associate with lysosomes, the endoplasmic reticulum, the Golgi apparatus, the plasma membrane, and endocytotic vesicles, corroborating a role for LRBA in polarized vesicle transport.^E14^ LRBA-deficient B cells show defects in autophagy and apoptosis.^E11,E16^

Some LRBA-deficient patients were reported to have early-onset primary hypogammaglobulinemia and reduced numbers of class-switched memory B cells,^E11^ suggesting a diagnosis of hyper-IgM syndrome or common variable immunodeficiency.^E17-E20^ Other patients initially presented with autoimmune cytopenia or inflammatory bowel disease but normal immunoglobulin concentrations.^E12,E13^ Overall, the hitherto 11 published patients present with a broad spectrum of symptoms, as summarized in Fig E1. Allogeneic HSCT as a treatment option in patients with refractory autoimmune disease is discussed controversially as either being considered a useful replacement of the immune system with the additional option of a beneficial concomitant “graft versus autoimmunity” effect or seen as too dangerous because of higher morbidity and mortality with uncertain curative potential.^E21-E25^

## Methods

### Flow cytometry

In addition to routine chemistry and clinical immunology laboratory analyses, special immunologic analyses included flow cytometry (fluorescence-activated cell sorting) performed on a Cytomics FC500 flow cytometer (Beckman Coulter, Brea, Calif) with a panel of mAbs from Beckman Coulter (Vienna, Austria), Becton Dickinson (Vienna, Austria), Dako (Glostrup, Denmark), Miltenyi Biotech (Vienna, Austria, and Bergisch Gladbach, Germany), as described previously.^E26^

### Functional and apoptosis assays

To test lymphocyte proliferation *in vitro*, mononuclear cells (1e5) from peripheral blood of healthy donors and patients were incubated with PHA (12.5 μg/mL; Sigma Chemicals, St Louis, Mo), soluble CD3 mAb (2 μg/mL; OKT3, Ortho, Raritan, NY), phorbol myristate (10^−7^ mmol/L, Sigma Chemicals), or medium in round-bottom 96-well culture plates for 72 hours, after which cells were pulsed with methyl-tritiated thymidine (1 mCi per well) for 18 hours and processed as described previously.^E27^ Apoptosis assays were performed, as described elsewhere.^E28^ Phagocytosis and oxidative burst were detected with dihydrorhodamine-123.

### Immunohistochemical analysis and *in situ* hybridization

Sections of formalin-fixed, paraffin-embedded tissue specimens were stained with mouse anti-human CD3 (clone F7.2.38; dilution 1:50; Dako, Glostrup, Denmark), mouse anti-human CD8 (clone C8/144B; dilution 1:30; Dako), and mouse anti-human CD4 (clone 4B12; dilution 1:20; Labvision, Fremont, Calif) mAbs, according to the supplier's specifications. *In situ* hybridization of EBV RNA was done with the INFORM EBER probe (Ventana-Roche, Mannheim, Germany), according to the supplier's specifications.

### Genetic analyses

Both affected subjects were genotyped on the Affymetrix GeneChip Human Mapping 250K Nsp Array at the Center for Medical Research at the Medical University of Graz. Autozygosity mapping was performed with dCHIP (http://biosun1.harvard.edu/complab/dchip).^E29^ Exome sequencing was performed, applying the Nextera Exome Enrichment Kit (Illumina, San Diego, Calif), according to the manufacturer's recommendations. In brief, 50 ng of genomic DNA were tagmented (tagged and fragmented) with Nextera transposase. This process simultaneously added adapter sequences, which were used as primer-binding sites for a limited-cycle PCR. After purification steps, the 12-plexed library was mixed with capture probes for enriching exonic regions and PCR amplified. Cluster generation was performed with the Illumina cBot Cluster Generation System according to the TruSeq PE Cluster Kit v3 (cBot-HS Reagent Preparation Guide, Illumina). Sequencing was performed on a HiSeq2000 (Illumina) applying the TruSeq SBS Kit v3-HS (200-cycles). Reads were demultiplexed with Casava. Alignment to the human genome hg-19 was done, applying the Burrows-Wheeler aligner. The Genome Analysis Toolkit was used to call single nucleotide and insertion/deletion variants.

### LRBA expression by means of fluorescence-activated cell sorting analysis

PBMCs were isolated from venous blood of a healthy donor, the heterozygous mother, and both patients with Lymphoprep (Axes-Shield Kabi Norge As, Oslo, Norway). After stimulation with 10 ng/μL PHA (L1668; Sigma, St Louis, Mo) for 72 hours at 37°C in a 5% CO_2_ atmosphere, intracellular expression of LRBA in unstimulated and stimulated PBMCs was measured by using flow cytometry. Briefly, cells were first permeabilized and fixated with BD Cytofix/Cytoperm solution (BD Biosciences, Heidelberg, Germany) and then stained with rabbit polyclonal anti-LRBA antibody (HPA019597; Sigma-Aldrich, Munich, Germany) for 30 minutes at 4°C. Subsequently, a phycoerythrin-conjugated secondary antibody against the LRBA antibody (PE[ab′]2 Donkey anti-rabbit IgG, reference 558416, BD Biosciences) was added and incubated for 30 minutes. Cells were then washed and analyzed on a FACSCanto II. Data analysis was performed with FlowJo software (version 7.6.5; TreeStar, Ashland, Ore).

## Results

### Further information on the clinical course and immune phenotype of 2 sisters with LRBA deficiency

#### Patient 1

Patient 1, a now 19-year-old girl of consanguineous Turkish parents of Kurdish origin presented with ITP at the age of 2 years, which was responsive initially to immunoglobulin treatment (Fig 1, *A*, and see Fig E1, *B*). The girl soon started to experience recurrent ear, nose, and throat and airway infections that required repeated courses of oral antibiotics. Starting at age 4 years, her weight gain decelerated, and generalized lymphadenopathy was reported (incipient LPD; Fig 1, *A*). The patient presented with relapsing pneumonia and pancytopenia at the age of 6 years. Additionally, she became dystrophic. At age 6 years, her weight was at the third percentile (16 kg), and her length was at the 10th percentile (110 cm). Hepatosplenomegaly, LPD, fatty stools, and diarrhea were noticed. Elastase in stool was 22 μgE1/μg (normal, 200-2500), indicating exocrine pancreas insufficiency. After repeated courses of IVIG treatment, we tested the vaccine response during an IVIG-free interval and found a good response to polio, *Haemophilus* species, and tetanus. Cytomegalovirus was detected in the urine, but anti-cytomegalovirus IgG levels were negative. Levels of gliadin-directed IgA and IgG and various autoantibodies, including phospholipid and smooth muscle antibodies, and Coombs test results were positive (Table I and see Table E1), indicating multiorgan autoimmunity. The pancytopenia responded well to reinitiation of IVIG therapy; chronic diarrhea was observed, and inflammatory disease was diagnosed clinically but never verified by means of gut biopsy. The clinical presentation suggested ALPS, and corresponding immunologic and genetic analyses were performed. Repeated analyses of CD3^+^CD4^−^CD8^−^TCRα/β^+^ (DNT) cells identified between less than 1% and 3% of CD3^+^ T cells at 6 to 8 years of age, and results of other cellular analyses, including apoptosis assays and mitogen stimulation *in vitro*, performed at that time were normal (Table I and see Table E1). DNA sequence analysis of *CD95*, *CD95L*, caspase 8, caspase 10, *SAP*, and *XIAP* showed no pathogenic mutation.

The conditioning regimen consisted of 5 × 30 mg/m^2^ fludarabine, 3 × 20 mg/kg ATG-F, and 2 × 70 mg/m^2^ melphalan (Fig 1, *A*). The bone marrow graft contained 5.5 × 10^6^/kg CD34^+^ stem cells and 4.2 × 10^7^/kg CD3^+^ T cells. Standard GvHD prophylaxis, consisting of cyclosporine A intermittently supplemented with corticosteroids and mycophenolate mofetil (MMF; Fig 1, *A*), was administered. The post-HSCT period was complicated by adenovirus viremia and hepatopathy, as well as suspected graft failure caused by pancytopenia (despite complete donor chimerism) potentially linked to viral reactivation and immunodysregulation around day +60, prompting a stem cell boost with growth factor support. This ultimately led to stable engraftment with 100% donor chimerism until today. A liver biopsy on day +123 after HSCT showed no signs of GvHD but was compatible with viral (HHV6 or adenovirus induced) or toxic damage. There were no other signs of GvHD. Antiviral therapy was intensified, and the regular post-HSCT immunosuppression (cyclosporin A, corticosteroids, and short-term MMF) was tapered. Liver parameters remained increased (between 3 and 10 times the upper limit of normal, predominantly alanine aminotransferase) and smooth muscle and anti-mitochondrial antibodies (especially of the M2 subfraction), which are often associated with primary biliary cirrhosis (PBC), were detectable (Table I and see Table E1). Four years after HSCT, treatment for suspected alloimmune/autoimmune hepatitis could be terminated based on normalized liver parameters, but the recurrence of ITP (platelet counts: 11,000-16,000/μL; constantly >100,000/μL since engraftment) led to short and unsatisfying attempts at intensified systemic immunosuppression with MMF and rapamycin. The direct Coombs test was not done at the reoccurrence of ITP because RBC and hemoglobin levels were normal; however, a recent evaluation showed negative direct Coombs test results and negative anti-platelet autoantibody levels (Table I). Furthermore, patient 1 had no detectable viral load at various occasions within the last 4 years.

Notably, recent laboratory investigations of the maternal donor performed 9 years after the HSCT of her daughter showed an identical pattern of anti-mitochondrial autoantibodies, suggesting a subclinical familial predisposition to PBC-associated autoantibodies.

The mother had a negative direct Coombs test result and negative antinuclear and borderline positive platelet antibody levels but not other autoantibodies or clinical evidence of autoimmunity like her daughters, such as vitiligo, immune cytopenia, or other diseases. She is under yearly observation at the hepatology department and has normal alkaline phosphatase and gamma-glutamyl transferase levels, and results of both liver function tests and liver ultrasound are unremarkable. Other heterozygous family members (the father and brother of patients 1 and 2) had negative test results for autoantibodies.

In patient 1 a second and a third liver biopsy were performed on days 200 and 330 after HSCT, showing progressive fibrosis with massive portal lymphocytic and histiocytic infiltration sparing bile ducts, as well as Kupffer cell and parenchymal (hepatocyte) siderosis compatible with chronic autoimmune hepatitis or viral/toxic damage but no signs typical for GvHD or biliary cirrhosis. No viral nucleic acid was detectable in liver parenchyma or blood on that occasion or later. Immunosuppression against autoimmune hepatitis, namely corticosteroids and azathioprine, was started (Fig 1, *A*) and slowly ameliorated hepatopathy. Only last year, the patient started to experience vitiligo, and topical steroid treatment was initiated, leading to stabilization of the disease. It is unclear whether this condition is an alloimmune response (ie, late chronic GvHD) or caused by the underlying autoimmune disease.

#### Patient 2

Patient 2 had been evaluated earlier during infancy as a potential stem cell donor, when RBC-directed and platelet antibodies were detected but B, T, and DNT cell numbers and additional cellular immunologic analyses were normal. Before treatment, her weight and body length were between the 90th and 97th percentiles. Notably, the patient's immune system never recovered from anti-CD20 treatment, with ongoing B-cell deficiency, complete lack of class-switched memory B cells, and profound hypogammaglobulinemia resembling common variable immunodeficiency (Table I) that required initiation of immunoglobulin substitution. Soon after the first clinical manifestation, patient 2 started to experience recurring urinary tract infections without anatomic predisposition, and recurrent virus-induced diarrhea began (norovirus, adenovirus, or both were detectable on various occasions; Fig 1, *B*).

Stomach biopsy specimens showed chronic gastritis in the gastric corpus, with apoptotic bodies within and apoptotic debris underneath the epithelium and focally enhanced chronic active gastritis in the antrum (Fig 2, *B-D*, and see Fig E2, *D-I*). Colonic biopsy specimens showed epithelial cell damage indicated by apoptotic bodies within and underneath the epithelium, as well as chronic mucosal damage indicated by moderate crypt distortion; sparse apoptotic bodies could be seen in the epithelium of the crypts; and abundant neutrophilic granulocytes within and migrating through capillaries within the lamina propria indicative of vasculitis were evident, although plasma cells were absent (Fig 2, *B-D*, and see Fig E2, *D-I*). EBV RNA was detected in several epithelial nuclei in the stomach, as well as duodenal biopsy specimens (see Fig E2, *H*). Malabsorption and growth arrest were observed, which did not improved with a gluten-free diet over 18 months or tube feeding with formula nutrition. After total parenteral nutrition (TPN) was commenced at 10 years of age, when body length and weight were at the third percentile, she restarted to thrive in parallel to the third percentile. Additionally, mild hepatopathy with increased transaminase levels but without signs of cholestasis started at 7.5 years of age and has persisted to date. Anti-mitochondrial antibodies but neither smooth muscle nor M2 antibodies were detectable; no liver biopsy has been performed. Albumin concentrations and levels of the bile duct enzymes alkaline phosphatase and gamma-glutamyl transferase have been normal. Various attempts at systemic or topical immunosuppression with corticosteroids or rapamycin to treat her enteropathy were of limited and/or short-term success. One episode of bilateral gonarthritis at the age of 9.5 years was noted, which responded to nonsteroidal anti-inflammatory drugs.

## Discussion

Here we report 2 patients harboring a novel mutation of *LRBA* (NM_001199282:c.7162delA; p.T2388Pfs*7). The patients presented with several severe symptoms, many of which have been described previously, such as cytopenias and lymphoproliferative syndrome (Fig 1, *A* and *B*, and see Fig E1),^E11-E13^ and some novel additional findings, such as exocrine pancreas dysfunction and intestinal vasculitis. The occurrence of primary hypogammaglobulinemia is controversial in patients with LRBA deficiency because this has been described only in some subjects.^E11^ Other patients, including the 2 patients treated at our institution, presented with normal immunoglobulin levels and antibody production before initiation of immunosuppressive treatment.^E12,E13^ Thus in summary, the phenotype of LRBA deficiency can include chronic diarrhea/inflammatory bowel disease; immune cytopenia; recurrent bacterial infections of the airways, ear/nose/throat, and other organs; and various other autoimmune/immune dysregulatory features, including arthritis, glomerulonephritis, gastritis, diverse dermatologic manifestations, lymphoproliferative disease, central nervous system granuloma (Fig 1, *A* and *B*, and see Fig E1), and hypogammaglobulinemia.

Currently described mutations in *LRBA* include large deletions, frameshift mutations that lead to a premature stop codon, and 1 C-terminal missense mutation.^E11-E13^ All of these mutations lead to an absence of protein expression. Our novel mutation was detected by using exome sequencing and is a single base pair deletion at position c.7162, causing a frameshift in exon 47 (p.T2388fs*7) and leading to a translational stop before the alpha-H helix of the BEACH domain in the C-terminal region of the protein,^E15^ likely inducing nonsense-mediated decay. This domain is involved in intramolecular, protein-protein, and protein-DNA interactions. Comparing the clinical symptoms of immunodeficiency and autoimmunity of our patients with those of the previously described patients with respect to their mutations and considering that in all patients LRBA protein is completely missing, it is not possible to draw any conclusion on a potential genotype-phenotype correlation.

Although certain refractory courses of systemic sclerosis, multiple sclerosis, systemic lupus, Crohn disease, and other autoimmune diseases are becoming a standard indication for autologous stem cell transplantation,^E30^ the indications for allogeneic HSCT in patients with autoimmune diseases remain controversial, given the risk of histoincompatibility reactions, such as GvHD, and the increased incidence of life-threatening infections in the allogeneic setting compared with autologous stem cell reinfusion. This debate is reflected by a number of reviews and meta-analyses on various autoimmune disease subgroups.^E21-E25^ In general, the agreement on HSCT as a possible therapy is higher in autoimmune diseases involving cytopenias than in those involving a single organ. With the exception of a certain number of cotransplanted peripheral memory and effector T cells, replacement of a deficient immune system through allogeneic HSCT results in a reset of tolerance-inducing mechanisms with newly arising naive T and B cells that undergo central and peripheral tolerance checkpoint editing. This implies that any autoimmune pathomechanism that arises from defective immune or blood-derived cells, including antigen-presenting cells, should be correctable.

The high degree of consanguinity within this family, as well as the HLA identity of the mother with both daughters, estimated a smaller amount of antigen disparity than in an unrelated donor allogeneic setting. To what extent the graft and sustained complete donor chimerism conferred a lasting cure for patient 1's PID remains to be seen; however, 9 years of follow-up after HSCT revealed a good partial remission with moderate cITP, vitiligo, and subclinical presence of M2 autoantibodies without any reduction in patient-reported quality of life. The fact that the LRBA-heterozygous maternal donor also has anti-mitochondrial antibodies without clinical symptoms of PBC like her daughters might have 2 implications.

First, perhaps the heterozygosity of the *LRBA* frameshift mutation in the mother associated with a reduction in LRBA protein concentration is pathomechanistically relevant. This has not been documented before because heterozygous relatives were silent carriers in previously described families,^E11^ although it is unclear whether all clinically healthy carriers were tested for the presence of any autoantibodies. In that case the allogeneic HSCT in patient 1 might resemble in part an autologous HSCT with a reset of the immune system and an ameliorated but not fully corrected LRBA deficiency.

Second, another familial predisposition toward PBC-associated autoantibodies than the LRBA deficiency of patients 1 and 2 exists that also affects the mother. PBC is more frequent in female than male subjects [9:1], and a familial predisposition is indicated by the highly increased risk in monozygotic twins^E31^ and association with *HLA-DRB1*08*, *HLA-DQB1*, and other loci, including *IL12A*, *IL12RB2*, *STAT4*, and *CTLA4*, respectively.^E32,E33^ In the presented family we found 3 heterozygous single nucleotide polymorphisms in 3 genes (rs3748816 in *MMEL1*, rs907092 in *IKZF3*, and rs2305480 in *GSDMB*), which were recently revealed to be associated with an increased risk of PBC in genome-wide association studies,^E32,E33^ suggesting the presence of an additional polygenetic predisposition toward M2 autoantibodies and potentially PBC in this pedigree apart from LRBA deficiency.

The younger sibling has been considered a candidate for stem cell transplantation since the first presentation after the experience of patient 1, but 3 factors have argued against it. First, it was suspected that the patient's bowel inflammation represented an unpredictable risk and might not benefit from HSCT given the chronic norovirus infection and near-total malabsorption. Second, an *LRBA* wild-type HLA-matched donor would be preferable instead of the HLA-identical mother, who was a heterozygous carrier of the *LRBA* mutation, but such a donor was not available. Third, combined HSCT and bowel transplantation with potentially only partial reconstitution of LRBA expression from the HLA-identical but LRBA-heterozygous mother appeared too experimental, given that under TPN, the girl attends school and showed near-normal physical development for the last 2 years. Nevertheless, any deterioration of her disease state would warrant a re-evaluation of the potential risks and benefits of HSCT.

Today, facing a total of only 13 published patients, including the 2 LRBA-deficient patients described here, it is unclear whether HSCT should be recommended generally on diagnosis. A high variation of the clinical severity is known from other PIDs with immunodysregulation, such as combined immunodeficiencies, immune dysregulation–polyendocrinopathy–enteropathy–X-linked syndrome, and CD27 deficiency.^E34-E38^ Although it appears probable that our patient 2 might have benefited from early HSCT and many of the previously described other clinical courses were severe, deriving a more comprehensive map of genotype-phenotype correlation and the natural course of LRBA deficiency requires longer follow-up and detection of more patients with different genotypes.

## References

[1] Alangari A., Alsultan A., Adly N., Massaad M.J., Kiani I.S., Aljebreen A. (2012). LPS-responsive beige-like anchor (LRBA) gene mutation in a family with inflammatory bowel disease and combined immunodeficiency. J Allergy Clin Immunol.

[2] Burns S.O., Zenner H.L., Plagnol V., Curtis J., Mok K., Eisenhut M. (2012). LRBA gene deletion in a patient presenting with autoimmunity without hypogammaglobulinemia. J Allergy Clin Immunol.

[3] Lopez-Herrera G., Tampella G., Pan-Hammarstrom Q., Herholz P., Trujillo-Vargas C.M., Phadwal K. (2012). Deleterious mutations in LRBA are associated with a syndrome of immune deficiency and autoimmunity. Am J Hum Genet.

[4] Gratwohl A., Baldomero H., Sureda A., Apperley J., Carreras E., Gluckman E., Masszi T. (2012). Indications for and current practice of allogeneic and autologous HSCT. The EBMT handbook—haematopoietic stem cell transplantation: European Group for Blood and Marrow Transplantation & European School of Hematology. European School of Hematology, Paris, France.

[5] Snowden J.A., Saccardi R., Farge D., Apperley J., Carreras E., Gluckman E., Masszi T. (2012). Indications for HSCT in adults—Autoimmune diseases. The EBMT handbook—haematopoietic stem cell transplantation: European Group for Blood and Marrow Transplantation & European School of Hematology. European School of Hematology, Paris, France.

[6] Canale V.C., Smith C.H. (1967). Chronic lymphadenopathy simulating malignant lymphoma. J Pediatr.

[7] Price S., Shaw P.A., Seitz A., Joshi G., Davis J., Niemela J.E. (2014). Natural history of autoimmune lymphoproliferative syndrome associated with FAS gene mutations. Blood.

[8] Seidel M.G., Urban C., Sipurzynski J., Beham-Schmid C., Lackner H., Benesch M. (2014). High response rate but short-term effect of romiplostim in paediatric refractory chronic immune thrombocytopenia. Br J Haematol.

[9] Seelow D., Schuelke M., Hildebrandt F., Nurnberg P. (2009). HomozygosityMapper—an interactive approach to homozygosity mapping. Nucleic Acids Res.

